# State of Research on Tissue Engineering with 3D Printing for Breast Reconstruction

**DOI:** 10.3390/jcm14196737

**Published:** 2025-09-24

**Authors:** Gioacchino D. De Sario Velasquez, Yousef Tanas, Francesca Taraballi, Tanya Herzog, Aldona Spiegel

**Affiliations:** 1 University of Maryland Medical Center, 22 South Greene St., Baltimore, MD 21201, USA; giodesario@gmail.com; 2 Institute for Reconstructive Surgery, Houston Methodist Hospital, Weill Cornell Medicine, Houston, TX 77030, USA; youseftanas@gmail.com; 3 Houston Methodist Research Institute, Houston, TX 77030, USA; ftaraballi2@houstonmethodist.org (F.T.); tlherzog@houstonmethodist.org (T.H.)

**Keywords:** tissue engineering, 3D printing, breast reconstruction, scaffold, polymer

## Abstract

**Background:** Three-dimensional (3-D) printing paired with tissue-engineering strategies promises to overcome the volume, contour, and donor-site limitations of traditional breast reconstruction. Patient-specific, bioabsorbable constructs could enable one-stage procedures that better restore aesthetics and sensation. **Methods:** A narrative review was conducted following a targeted PubMed search (inception—April 2025) using combinations of “breast reconstruction,” “tissue engineering,” “3-D printing,” and “scaffold.” Pre-clinical and clinical studies describing polymer-based chambers or scaffolds for breast mound or nipple regeneration were eligible. Data was extracted on scaffold composition, animal/human model, follow-up, and volumetric or histological outcomes. **Results:** Forty-three publications met inclusion criteria: 35 pre-clinical, six early-phase clinical, and two device reports. The predominant strategy (68% of studies) combined a vascularized fat flap with a custom 3-D-printed chamber to guide adipose expansion. Poly-lactic acid, poly-glyceric acid, poly-lactic-co-glycolic acid, poly-4-hydroxybutyrate, polycarbonate, and polycaprolactone were the principal polymers investigated; only poly-4-hydroxybutyrate and poly-lactic acid have been tested for nipple scaffolds. Bioabsorbable devices supported up to 140% volume gain in large-animal models, but even the best human series (≤18 months) achieved sub-mastectomy volumes and reported high seroma rates. Mechanical testing showed elastic moduli (5–80 MPa) compatible with native breast tissue, yet long-term load-bearing data are scarce. **Conclusions:** Current evidence demonstrates biocompatibility and incremental adipose regeneration, but clinical translation is constrained by small sample sizes, incomplete resorption profiles, and regulatory uncertainty. Standardized large-animal protocols, head-to-head polymer comparisons, and early human feasibility trials with validated outcome measures are essential next steps. Nevertheless, the convergence of 3-D printing and tissue engineering represents a paradigm shift that could ultimately enable bespoke, single-stage breast reconstruction with superior aesthetic and functional outcomes.

## 1. Introduction

Breast cancer presents an important challenge to global healthcare and significantly impacts on the quality of life of countless woman, necessitating advancements in treatment and post-surgical care. Within this framework, breast reconstruction emerges as a beacon of hope, offering a path to regain physical and emotional well-being. While substantial strides have been made in reconstruction techniques, a gap persists in accessibility, affordability, and safety, underscoring a pressing need for innovation and improvement.

In 2020, the U.S. reported 239,612 new female breast cancer cases with 42,273 deaths, equating to 119 new cases and 19 deaths per 100,000 women [1]. Beyond its devastating health impact, breast cancer imposes a significant financial burden on the U.S. healthcare system. A recent nationwide study revealed that cancer patients experience almost 4 times higher mean expenditures per person ($16,346) compared to those without cancer ($4484) [2]. This financial strain is mirrored in the field of breast reconstruction, where the costs of current methods, coupled with the need for specialized surgical skills, may sometimes place these critical procedures difficult to obtain for patients who lack insurance coverage or must travel long distances to microsurgical centers. Although breast reconstruction is generally covered by U.S. insurance plans (including after passage of the WHCRA) gaps in access persist for uninsured or under-insured patients and in settings where surgeons decline Medicaid/Medicare or charity cases.

Moreover, the burden of breast cancer transcends beyond the numbers, profoundly affecting women’s quality of life. Survivors often struggle in their cognitive, sexual, and emotional well-being, underscoring the broader challenges that come with recovery [3]. In this context, breast reconstruction after mastectomy is a key element in the journey towards recovery, improving emotional functioning and social functioning scores, especially in younger patients [4].

Current options—alloplastic and autologous—each carry their own set of complications. Alloplastic or implant-based breast reconstruction can lead to complications such as capsular contracture, implant failure, rupture, and cancer [5]. The FDA has reported new cases of cancers, including squamous cell carcinoma and lymphomas, in the capsule surrounding breast implants, distinct from the previously recognized Breast Implant-Associated Anaplastic Large Cell Lymphoma (BIA-ALCL) [6,7].

Alternatively, autologous breast reconstruction has been associated with higher satisfaction compared to implant-based reconstruction [8]. Still, patients with autologous breast reconstruction may suffer complications including flap failure, mastectomy flap necrosis, donor site morbidity, and emergent reoperations [9,10]. Further, autologous breast reconstruction is often challenging, requiring highly trained microsurgeons and microsurgery-equipped hospitals, which are often lacking in developing countries [11,12].

Autologous fat grafting for breast reconstruction is globally accepted, with its safety and efficacy confirmed by many clinical studies [13,14]. Nevertheless, fat grafting for breast reconstruction is constrained by the limited volume it can provide and the inconsistent retention rates, often necessitating multiple sessions to attain a satisfactory final volume [15,16].

With the challenges posed by conventional methods, the medical community has eagerly sought innovative solutions. The fields of tissue engineering and 3D printing are two domains that have shown promise in revolutionizing medical treatments across various sectors [17,18]. These innovative methods offer the possibility of reconstructions tailored to an individual’s anatomy, using the patient’s own cells and host response towards tissue regeneration, thereby promising better functional and aesthetic outcomes [19].

With the ability to customize the structure, composition, and mechanical properties of biomaterials, 3D printing has opened doors for the development of implants that mimic the natural extracellular matrix, facilitating cellular integration and tissue regeneration [20]. Furthermore, such advancements might pave the way for improved functional and aesthetic outcomes in breast reconstruction.

This narrative review focuses on the latest developments in tissue engineering and 3D printing for breast reconstruction. We aim to identify current research gaps, guide future studies in this domain, and critically evaluate the potential of these emerging technologies to transform breast reconstruction procedures in medical practice.

## 2. Biomaterials and Tissue Bioengineering for Breast Reconstruction

### 2.1. The Current State of Research on Reconstructive Materials

The field of tissue engineering has seen a noticeable surge recently, particularly in the development of ideal biomaterials for breast reconstruction. In the past, substances like hydrogels, ceramics, and biopolymers showed great promise for fostering cellular growth and directing tissue rejuvenation [21]. The primary objective of research has been to develop materials that mimic the extracellular matrix found naturally in breast tissue while promoting cell adhesion, development, and specialization [22,23].

Tissue engineering combines cells, biomaterials, and advanced methodologies to develop biological structures that both mirror and augment the inherent functionalities of human organs and tissues [24]. Over time, this domain has significantly advanced, now emphasizing not just the regeneration of in vivo tissues without innate self-repair capabilities, but also the creation of in vitro models that illuminate cellular dynamics [25,26,27,28]. These models also provide platforms for cutting-edge applications like organs-on-a-chip and medication screening [29,30].

In the world of 3D printing microvascular networks, current methodologies exhibit limitations in precisely emulating the native cellular composition and functionality of vascular structures. Additionally, these techniques often lack the capacity to control hierarchical dimensions accurately [31]. Consequently, the complete replication of native microvascular networks via 3D printing remains unfeasible at this juncture.

### 2.2. Role of Biodegradable Materials in Tissue Engineering

Biodegradable materials are of particular interest in tissue engineering for breast reconstruction. Over time, a process of gradual degradation ensues, concomitantly with the progressive integration of native tissues during the regenerative phase, removing the necessity for subsequent surgical intervention aimed at implant removal. This feature allows the preservation of the regenerated tissue’s structural integrity while minimizing the long-term complications linked to non-biodegradable components [32]. The degradation rate, however, must be carefully tuned to match the rate of tissue regeneration [33].

### 2.3. Advantages and Limitations of Biodegradable Materials

While biodegradable materials present an exciting frontier for breast tissue engineering, striking a balance between their evident benefits and inherent challenges remains critical. One key advantage is that, unlike traditional implants, they are designed to be reabsorbed by the body, avoiding the consequences of long-term inflammation of permanent implants [34,35].

Although surgical insertion of biodegradable materials can still lead to surgical infections, researchers have suggested that biodegradable materials carry a lower risk [36]. The degradation rate of these materials needs careful calibration to provide sufficient support without triggering complications. Other concerns include the potential loss of mechanical strength over time and the long-term impact of degradation byproducts in the body [37].

## 3. Preclinical Studies on Reconstructive Materials with Tissue Engineering

### Materials and Methods

We performed a review of the available literature of preclinical research in tissue engineering for breast reconstruction in PubMed on 16 April 2025 using the following search strategy: “(“Mammaplasty”[Mesh]) AND “Tissue Engineering”[Mesh].” The search yielded 39 results published between 2001 and 2025, and 10 articles were deemed relevant and are reviewed below. Another search was performed to review the current state of research, especially for 3D printing tissue engineering methods for breast reconstruction. The search was conducted in PubMed on 20 August 2023 using the following search strategy: (“Printing, Three-Dimensional”[Mesh]) AND (“Mammaplasty”[Mesh]). This time, the search resulted in only 9 results, and only 2 were found relevant and were targeted to nipple reconstruction.

Overview of preclinical research in tissue engineering for breast reconstruction (Table 1).

As breast reconstruction techniques evolve, preclinical studies have seen significant advancements [38,39,40,41,42,43]. Nonetheless, when delving into tissue bioengineering specific to breast reconstruction, there is a noticeable scarcity of published research.

**Table 1:** Overview of preclinical research in tissue engineering for breast reconstruction.

Author, Year	Study Objective	Methodology	Materials Used	Key Findings	Limitations
[44]	Examine co-culture of mammary cells and adipocytes in 3D collagen	Human mammary epithelial cells and preadipocytes co-cultured in 3D collagen gel matrix ^†^	Collagen gel matrix	Both cell types expanded through multiple subcultures, maintained normal cell distribution and growth patterns	Limited to in vitro environment
[45]	Enhance adipocyte survival for lipo-injection	Selective in vitro culturing of preadipocytes ^†^	Preadipocytes	Increased proliferation and survival in cell cultures	Limited to in vitro environment
[46]	Study stromal-epithelial interactions	Cocultures of human mammary epithelial cell line (MCF10A) and human mammary fibroblasts embedded in type I collagen or mixed Matrigel-collagen matrix ^†^	MCF10A, fibroblasts, type I collagen, Matrigel-collagen matrix	Formation of ductal and alveolar structures confirmed histologically	Limited to in vitro environment
[47]	Upscale small-animal adipose tissue-engineering models to a large animal (pig)	Large-volume (78.5 mL) subcutaneous chambers enclosing fat flap in pigs ^‡^	Dome-shaped perforated polycarbonate TEC, poly(L-lactide-co-glycolide) sponge	Significant fat flap growth up to 56.5 mL from initial 5 mL by 22 weeks	Limited translation to human models
[48]	Evaluate longevity of tissue-engineered adipose tissue	Chambers implanted in mice groins, filled with Matrigel and heparin; varied configurations (autograft, open, fat flap) ^‡^	Matrigel, heparin, autologous fat	Higher adipose tissue volumes and vascularization, especially in fat flap group	Animal model; limited human applicability
[49]	Generate adipose tissue from vascularized fat flap inside a chamber	Rat model, chambers with or without PLGA scaffolds ^‡^	Polycarbonate chambers, PLGA scaffolds	Significant adipose volume increase in all chamber groups	Animal model; unclear mechanism for human scaling
[50]	Evaluate long-term stability of chamber-generated adipose tissue	Rat model, perforated vs. nonperforated chambers ^‡^	Polycarbonate chambers	Volume growth, greater in perforated chambers	Animal model limitations, unclear scalability to humans
[51]	Assess external suspension device for adipose tissue growth	Rabbit model, external suspension vs. traditional chamber ^‡^	External suspension device (negative pressure)	Larger volume growth with external suspension (81 mL vs. 31 mL over 36 weeks)	Animal model, device usability in human scenarios unclear
[52]	Effects of irradiation on fat flap growth	Rat model, bioresorbable PLGA-based TEC implantation; irradiation pre- or post-implantation ^‡^	PLGA-based bioresorbable TEC	Radiation reduced fat flap growth, introduced fibrosis and histological changes; viable as adjunct in breast reconstruction despite irradiation	Animal model; limited clinical translation
[53]	Influence of TEC design on adipose tissue growth	Rat and pig models, TECs (perforated vs. nonperforated), 3D-printed bioresorbable scaffolds ^‡^	PLA (rat), PGA (pig) scaffolds	Perforated TEC superior, rapid adipose growth, bioresorbable TEC achieved >140% volume growth in pigs	Animal models; unclear full clinical translation potential
[54]	Evaluate nipple projection retention using 3D scaffolds	Nude rat model, 3D-printed scaffolds filled with human cartilage ^‡^	3D-printed P4HB scaffolds, human costal cartilage	Improved nipple projection and tissue growth, regenerative response	Small animal model; uncertain scalability
[55]	Preserve nipple geometry using scaffolded cartilage	Nude rat model, external scaffolds with autologous cartilage ^‡^	3D-printed PLA external scaffolds, autologous cartilage	Maintained superior nipple volume, viable cartilage tissue with biomechanical similarity to human nipples	Animal model; limited human applicability
[56]	Enhance fat graft retention with scaffold support	Nude mice model, fat graft injected into scaffold ^‡^	3D-printed polycaprolactone scaffolds	Improved graft retention, angiogenesis observed; superior cellular preservation initially	Short-term animal study
[57]	Scaffold pre-vascularization for breast reconstruction	Minipig model, pre-vascularized scaffold compared to immediate grafting ^‡^	Polycaprolactone scaffolds	Pre-vascularized scaffolds improved adipose tissue retention significantly	Limited animal study duration, scalability unclear
[58]	Hybrid scaffold approach to improve fat graft survival	Male mice model, hybrid devices combining implants + scaffolds + inguinal fat grafts ^‡^	Polycaprolactone scaffolds, electrospun nanofibers, silicone implants	Improved adipocyte morphology at early stage; limited overall retention benefits	Small animal model; unclear human translation

Legend: ^†^ = in vitro study, ^‡^ = in vivo study.

Our first search revealed several efforts aimed at understanding and enhancing tissue growth in vitro. Early work by Huss et al. showed that co-culturing human mammary epithelial cells with pre-adipocytes in 3-D collagen supports breast-like tissue formation; a follow-up study found selectively expanded pre-adipocytes proliferated and survived better, suggesting a route to improve fat-graft retention. Krause et al. extended this by culturing mammary epithelial cells with fibroblasts in a 3-D matrix, producing ductal-alveolar structures within weeks. Subsequent in vivo studies shifted to polycarbonate tissue-engineering chambers: in a porcine model, a 5 mL groin fat flap placed in a perforated dome chamber grew to ≈56 mL over 22 weeks, and the volume remained stable for another 22 weeks after chamber removal, even when the construct was transposed to a submammary pocket [44,45,46,47].

Although most of these pre-clinical studies were not explicitly framed around breast reconstruction, their data are highly transferable. In mice, three Matrigel-filled chamber designs—autograft, open, and fat-flap—were followed for a year: chambers contiguous with vascular fat (fat-flap) generated the largest, well-vascularized adipose masses, while autografts showed more fibrosis and all configurations contained significantly more fat than at 6 weeks. In two rat experiments, Dolderer et al. implanted pedicled fat flaps in solid or perforated polycarbonate chambers (with or without PLGA scaffold); every chambered flap gained volume over 20 weeks, perforated shells outperformed solid ones, and ungrafted controls remained static. Newly formed tissue was predominantly vascularized fat and connective stroma, with minimal glandular elements [48,49,50]. Manufacturing details for the polycarbonate TECs were omitted, though PLGA scaffolds were made by thermally induced phase separation.

Jinlin et al. replaced TECs with an external negative-pressure suspension device: in rabbits, flap volume rose from 5 mL to 81 mL in 36 weeks (vs. 31 mL with chambers) while maintaining similar histology. At Lille, 3-D-printed PLGA TECs in rats tolerated irradiation, but post- or pre-implant radiation curtailed fat growth and increased fibrosis, whereas non-irradiated controls enlarged flaps substantially. TEC design also matters: in rats, perforated PLA shells grew fat 3–5× faster than solid ones, and in pigs a bioresorbable PGA TEC boosted flap volume > 140% by day 90 without systemic inflammation. Despite these advances, chamber-based approaches still yield volumes below full breast dimensions, limiting clinical applicability for larger reconstructions [49,50,51,52,53]. From our second search, only two studies were found to be relevant, both targeting nipple reconstruction. A 1 × 1 cm domed P4HB nipple scaffold packed with minced costal cartilage kept projection and volume for 6 months in nude rats; an internal lattice sped scaffold resorption [54]. Using a PLA sleeve around processed costal cartilage similarly preserved nipple size and native-like mechanics at 3 months [55]. Bao et al.’s 1.5 mm porous PCL mesh boosted fat-graft volume retention and early angiogenesis in mice over 8 weeks [56]. Together, 3-D-printed scaffolds clearly outperform traditional nipple or fat-graft methods [54,55,56]. Pre-vascularization helps large grafts: in minipigs, 75-cc PCL hemispheres left empty for 2 weeks then fat-filled showed 48% adipose area vs. 40% when fat was injected immediately, illustrating center-out angiogenic limits [57]. A mouse study of a 3-D-printed PCL sleeve coated with electrospun nanofibers on silicone implants similarly found better early adipocyte morphology—though capsule thickness and volume retention were unchanged—emphasizing ECM-like surface cues [58]. Evaluation of biodegradable materials in preclinical models: biocompatibility, degradation kinetics and biomechanical properties (Table 2).

Biocompatibility is a common concern in tissue engineering; hence, studies must ensure that introduced materials interact safely with host tissues. For breast reconstruction and other medical uses, materials must be both physiologically and immunologically compatible to prevent adverse reactions and ensure natural integration.

Many biomaterials used in medical implants and regenerative medicine often develop foreign body reactions upon implantation [59]. The foreign body reaction to biomaterials progresses through five phases: protein adsorption, acute and chronic inflammation, and the formation of foreign body giant cells and fibrous capsules [60].

Biodegradable materials are promising because they allow the creation of devices with the ability to provide temporary structure and mechanical support as the tissue regenerates until device resorption, minimizing long-term foreign body presence [61].

Much has been researched about the use of biodegradable materials for drug delivery systems [62], orthopedic devices [63,64], stents [65], and wound healing [66,67,68]. However, only a few bioabsorbable polymers have been tested for breast reconstruction, including PLA, PGA, PLGA, P4HB, and poly(d,l)-lactide polymer.

PLA is an aliphatic polyester with degradation products of lactic acid, typically degrading over 6 to 12 months. It is prized for good mechanical properties but can be brittle and produce inflammatory acidic products upon degradation [69,70]. PGA is used widely in sutures and degrades into glycolic acid within weeks to a few months. It is recognized for its strength and biocompatibility, though its quick degradation can sometimes pose challenges [71,72]. PLGA, a mix of PLA and PGA, degrades to release both lactic and glycolic acids over weeks to several months. It is versatile compared to PGA and PLA since the composition ratio can control its resorption time [73]. P4HB is known for flexibility and strength, degrading into 4-hydroxybutyric acid in about 12 to 18 months; however, unlike other resorbable polyesters such as PLA, PGA, and PLGA, its production is complex since it is exclusively synthesized in the fermentation process; therefore, it is less readily available and more costly [74,75]. Finally, Poly(D, L]-lactide combines two PLA stereoisomers and shares a similar degradation rate and product with PLA; its blend ratio influences its properties but can produce inflammatory products [76,77].

The degradation rate of biomaterials plays a pivotal role in determining the success of tissue regeneration. For breast reconstruction, it is paramount that the material degrades at a rate that allows the concurrent growth and maturation of the new tissue, ensuring the maintenance of structural integrity. Rapid degradation could lead to tissue collapse and inadequate support. In contrast, slow degradation might hinder natural tissue formation, causing prolonged foreign body reactions or fibrotic encapsulation.

A developmental scaffold should be biocompatible with controlled degradation, have a 3D interconnected pore design, offer structural support, and promote positive cell interactions [78].

One common issue found in the preclinical studies for breast reconstruction using 3D printing is the mechanical properties of the TEC or scaffolds per se, as they are made of stiff materials, which do not align with the mechanical properties of the breasts.

Breast tissue, being highly vascular and glandular, has specific needs for elasticity, sensation, and aesthetics [79]. Therefore, biodegradable materials for breast reconstruction should ideally emulate the biomechanical properties of native breast tissue to meet patients’ needs.

- Gaps in preclinical testing: lack of specific preclinical studies on reconstructive materials for breast reconstruction and Implications.

The rapid advancements in reconstructive surgery, particularly breast reconstruction, are commendable. However, a significant concern arises from the lack of comprehensive preclinical studies targeting reconstructive materials. Without abundant reproducible and robust preclinical research, the understanding of how these materials might interact within the body remains limited, keeping the door closed to potential unanticipated outcomes.

The implications of this data gap hinder clinicians from understanding the safety and the overall behavior of the materials and their consequences. Biocompatibility and integration with native tissues are primary concerns when implanting any new reconstructive material. The body’s response must be gauged to predict long-term outcomes [80,81].

Beyond safety, the performance of these materials, like their aesthetic results or longevity, remains unpredictable. Ethically, exposing patients to potential risks without comprehensive prior testing challenges the medical principle of “do no harm” [82,83].

The varied levels of success rate documented in preclinical studies for breast reconstruction regarding the utilization of tissue-engineered scaffolds or chambers, whether 3D printed or not, as highlighted in earlier cited studies, amplifies a critical issue of publication bias. The underrepresentation of studies yielding negative results could lead to the misallocation of funding in project investments and obstruct the exploration of alternative, potentially more effective approaches for breast reconstruction [84].

In essence, while innovation in breast reconstruction is crucial, it must be underpinned by rigorous preclinical testing to ensure superior outcomes, maintain ethical standards, and empower informed decision-making.

## 4. Clinical Indicators for Reconstructive Materials

### 4.1. Identification and Evaluation of Clinical Indicators of Success for Breast Reconstruction

Clinical indicators are measurable metrics used to assess the quality and outcomes of healthcare services. These can relate to the structure, process, or results of care. They serve as benchmarks that guide healthcare professionals and organizations in enhancing care quality. They must be valid, sensitive, and clearly defined to gauge healthcare performance effectively. While optimal indicators are evidence-based, some may be based on professional consensus [85].

The gold standard for evaluating clinical outcomes in breast reconstruction is the use of patient-reported outcome instruments such as the BREAST-Q, which is widely used to assess the impact of breast surgeries on patient satisfaction and quality of life [86,87,88]. However, because it relies on patient input, it cannot be applied in preclinical research, highlighting a key limitation in bridging preclinical models with patient-centered outcomes. Currently, there is no standardized objective measurement tool for breast reconstruction assessment [89]. Some surgeons rely on the overall assessment of objective indicators to assess reconstruction success. These indicators include survival, complications, and aesthetic outcomes such as breast symmetry, volume, color differences, scar appearance, and nipple-areolar complex [89,90,91].

Regarding preclinical research of reconstructive materials, most studies primarily focus on assessing fat volume retention/growth. However, there is oversight of some other aesthetic indicators other than volume, such as symmetry, color differences, and scar appearance.

On the other hand, bioabsorbable materials hold a significant advantage of their potential to gear toward 1 stage surgery, consequently decreasing the hospital burdens and exposure to the inherent risk of surgical procedures.

### 4.2. Assessment of Existing Clinical Studies on Reconstructive Materials (Table 3)

The need for new methods in breast reconstruction has prompted significant research contributions on the clinical front.

One study introduced a method combining a three-dimensional absorbable mesh construct, referred to as the “Lotus scaffold”, with autologous fat grafting. A “Lotus” 3-D absorbable mesh scaffold plus 50–100 cc fat graft was used to reconstruct 28 breasts in 22 patients (19-month mean follow-up). Three FDA-approved meshes were tested (TIGR, SERI, PHASIX). Patients averaged two further graft sessions (≈458 mL total). Histology showed a strong fibrotic response around TIGR but more organized adipose with PHASIX; the scaffold stayed highly elastic. Adverse events occurred in 25% (one subdermal cancer recurrence), yet nearly all surveyed patients rated their breasts soft and natural [92].

Other authors explored breast reconstruction using dome-shaped acrylic chambers with perforated walls and internal capacities ranging from 140 to 360 mL. In this study, 5 participants underwent thoracodorsal artery perforator (TAP) flaps, with volumes ranging from 6 to 50 mL. These flaps were placed inside the TEC. Patients were then monitored post-surgery for up to six months until chamber removal, except for 1 patient showing notable tissue growth, who received follow ups for 6 additional months which resulted in filling a 210 mL space. Three other patients exhibited no tissue growth beyond the initial flap’s dimensions, resulting in silicone implant reconstructions. Lastly, one patient had her chamber removed early due to discomfort. Histological analyses after chamber removal confirmed the presence of viable, well-vascularized fat inside the chamber for certain patients [93]. Notably, patient-reported and aesthetic outcomes were not assessed in this study.

This same approach is being studied by the clinical trial NCT05460780, which aims to assess the safety and efficacy of Matisse^®^, a TEC implant-based method for immediate breast reconstruction in Georgia (country). This method, however, involves a bioabsorbable TEC implantation with a pedicled LICAp or LTAp flap within it to support a flap growth [94]. Although no preliminary results have been published, a recent press report released in 2022 claimed that they achieved the first successful breast reconstruction with their device [95].

Other tools have been developed and tested in the field of breast reconstruction with 3D printing, such as surgical meshes, to provide breast support for implants and tissue expanders. One study investigated the outcomes of using SERI® Surgical Scaffold (Sofregen; Medford, MA, USA) conducted in The Netherlands. This retrospective study included 16 patients (22 breasts) and found no intraoperative issues. However, postoperative complications such as bleeding (5%), seroma (45%), and infection (9%) were observed. Significantly, 14% lacked scaffold integration, resulting in skin ulcerations. The authors also conducted a systematic literature review, pinpointing the scarcity and potential bias in existing studies, with many authors affiliated with the product’s producer [96].

Another clinical trial (NCT05437757) investigates an approach for breast reconstruction where patients’ fat tissue is harvested using liposuction and then injected into 3D printed scaffold implants made of polycaprolactone, a material approved for skull bone restoration by Australian regulatory authorities. Currently, the trial is seeking around 20 participants, primarily to determine the safety and efficacy of this approach [97].

Some patient-oriented concerns when assessing the TEC or scaffolds used for breast reconstruction are the biomechanical properties of the materials. Since these TECs provide a hard shell to enhance flap growth, they must maintain their mechanical properties for an acceptable period. However, such properties could lead to discomfort and unnatural breast shapes for relatively long periods, discouraging patients from undergoing this type of reconstruction. Indeed, in Morrison et al.’s [93] study, one out of 5 subjects underwent early removal of the TEC due to discomfort [93].

## 5. Discussion

Current trends and advancements in tissue engineering and 3D printing.

The increasing popularity and advancements in 3D printing technology have ushered in a new era of tissue engineering. The latest 3D printers offer improved precision, allowing for the creation of more complex tissue structures.

In addition, 3D bioprinters have emerged as a promising tool for tissue engineering. Three-dimensional bioprinting uses stem cells and bioinks to create 3D structures. These structures eventually integrate with a patient’s tissue, thanks to the bioinks’ support for cell growth and adhesion [98]. One limitation of bioprinters is the high costs, with prices ranging from $5000 to over $1,000,000 [99]. However, many conventional low-cost 3D printers have been proven to be able to shift to bioprinters by modifying some factors [100,101,102].

There is still a considerable journey ahead in research involving bioprinters. While bioink has been utilized to construct various breast cancer models [103,104], its application in the context of breast reconstruction remains unexplored.

### 5.1. Identification of Research Gaps and Areas for Future Exploration

This paper has already delved into the research gaps. It goes without saying that given the relatively emerging nature of this field, there exists a vast array of unexplored territories.

Artificial Intelligence has the potential to revolutionize 3D printing in healthcare by precisely adapting designs to complex body structures using sensory data, making real-time adjustments during the printing process, and predicting and adapting to rapid changes, like organ movements [105,106]. In breast reconstruction, AI could enable the creation of more tailored implants and offer real-time adaptability to patient-specific anatomies, enhancing the overall precision and outcomes of the procedure.

Another field that needs to be explored, both in the preclinical and the clinical phases, is the use of growth factors and mesenchymal stem cells that can aid in fat growth and replication to expand fat flaps and fat grafts. However, contrary to the philosophy of 3D printing in healthcare, which promises simplicity, this would add further steps and obstacles, including concerns about the oncological potential of fat grafts.

Moreover, it remains to be determined whether the implantation of 3D printed devices, based on each design and polymer, might interfere with monitoring breast cancer recurrence.

### 5.2. Regulatory Considerations and Future Perspectives

Research has been focused on simplifying breast reconstruction through 1 or 2-stage reconstructions using specific materials. However, the properties of these scaffolds are yet to be improved to achieve mechanical properties resembling natural breasts, allowing for comfortability and wellness of patients during the first months before the polymer reabsorbs. To attain this goal, more materials and designs for breast TEC or scaffolds need to be tested.

Further limitations regarding materials that may be used for breast reconstruction arise based on FDA regulations. The FDA’s Center for Devices and Radiological Health regulates medical devices in the U.S., including those created using 3D printing. Based on regulatory control level, devices are categorized into Class I, II, and III. Most Class I medical devices are exempt from Premarket Notification 510(k), whereas Class II devices usually require it, and Class III devices, the highest risk category, need Premarket Approval (PMA) [107,108]. For a device to gain FDA clearance through 510(k), it must demonstrate substantial equivalence to a predicate device that is legally marketed [109]. Currently, breast implants are classified as Class III devices [110]; furthermore, since the FDA has not yet approved or cleared any devices utilizing tissue bioengineering methods for breast reconstruction, new devices for this purpose will automatically require the more stringent PMA process before they can be legally marketed in the US.

In 2016, the FDA introduced draft guidance for 3D printed devices, providing advice on design, manufacturing, and testing. This guidance, still under review, details technical requirements and information expectations for premarket submissions [107].

The future of breast reconstruction with 3D printing methods envisions a scenario where the patient’s breasts are imaged, and the corresponding implants are manufactured directly within the healthcare facility, ensuring rapid availability at reduced costs. To support Point of Care manufacturing, the FDA is currently exploring regulatory frameworks for 3D printing of medical devices at the Point of Care. This initiative involves gathering stakeholder feedback to address the unique challenges of integrating 3D printing technologies in healthcare settings, focusing on managing risks and ensuring safety and effectiveness [111].

The regulatory landscape for 3D printing in breast reconstruction is intricate. While 3D printing offers tailored solutions vital for individual patient needs, it challenges traditional FDA frameworks designed for standardized devices. Balancing innovation and safety is critical. Defining responsibility becomes complex as 3D printing blurs the lines between manufacturers and healthcare providers. Transparent communication between innovators and regulatory bodies is crucial to navigating these challenges.

### 5.3. Challenges and Requirements for Clinical Translation

Challenges in clinical translation from animal models to humans in breast reconstruction arise due to the inherent biological differences between species, especially in tumor development and physiology [112]. Animal surgical models demonstrate limited success in translating to human clinical research, emphasizing an urgent need to explore alternative surgical research models.

Successful procedures in animal models might need significant modifications when applied to the larger and complex human anatomy. These changes are essential to ensure long-term outcomes, safety, and efficacy.

Concerning translational research in breast reconstruction using 3D printing, most studies perform reconstructions in healthy animal models, disregarding the impact of breast cancer resection, chemotherapy, and radiotherapy in such procedures. In contrast, studies in clinical trials are usually performed on patients immediately after cancer resection.

Further, objective evaluation of fat-flap and lipofilling outcomes is pivotal to the clinical translation of 3-D-printed, tissue-engineered constructs. The recent narrative review by Bogdan et al. [113] confirms that magnetic-resonance imaging (MRI) remains the quantitative gold standard for breast volumetry, yet advocates a pragmatic multimodal algorithm that layers MRI with high-resolution 3-D surface scanning, CT, dual-energy X-ray absorptiometry (DEXA), high-frequency ultrasonography with 2-/3-D shear-wave elastography, bio-impedance and even caliper measurements to balance accuracy, cost and accessibility. Crucially, the same review shows that seemingly minor variables (respiratory phase, posture, menstrual cycle and body-mass-index fluctuations) can shift measured breast volume by up to 8%, emphasizing the need to standardize or record these factors prospectively.

#### Clinical Translation and Implementation

Tissue engineering and 3D printing for breast reconstruction may face practical challenges in real-world applications. The cost of biomaterials remains a known barrier. Limited access to specialized grafting techniques may or may not pose significant limitations, as procedures such as liposuction and fat grafting are already feasible in many clinical settings; however, if future tissue-engineered strategies evolve to require complex, highly technical approaches, these may prove unfeasible in low-resource environments.

It is technically possible to manufacture and distribute standardized scaffolds from centralized facilities similar to how breast implants are currently produced and shipped. In contrast, the ability to design, customize, and fabricate patient-specific constructs locally would remain unfeasible in many low-resource settings. Regulatory variation across countries, shortages of trained personnel, and the lack of reimbursement frameworks add further complexity. These challenges underscore the need to consider not only innovation but also accessibility and scalability in the development of future clinical applications.

Taken together, 3-D-printed chambers/scaffolds for breast reconstruction remain investigational rather than practice-ready. Human data are limited and heterogeneous: the composite “Lotus” scaffold (absorbable mesh plus staged fat grafting) required ≈2 additional graft sessions (~458 mL total) with adverse events in ~25% despite favorable feel [92]; an acrylic tissue-engineering-chamber case series achieved substantial fill in only one of five patients and included early removal for discomfort [93]; and a SERI™ scaffold cohort reported frequent complications (e.g., seroma 45%, integration failure 14%) [96]. Two feasibility trials are in progress—the bioabsorbable MATTISSE^®^ TEC and scaffold-guided fat injection using PCL—so durability and acceptability remain to be demonstrated [94,95,97]. Implementation should prioritize patient comfort and revision burden while standardizing outcomes: MRI-based volumetry as the quantitative gold standard, complemented by accessible modalities, and co-primary patient-reported outcomes (e.g., BREAST-Q) [86,87,88,89,90,91,113]. Seemingly small methodological factors (posture, respiration, menstrual cycle, BMI) can shift measured volume by up to ~8%, so they should be prespecified and controlled [113]. Context matters clinically: irradiation curtailed fat growth and increased fibrosis in PLGA-TEC models, informing eligibility and timing in early studies [52]. Finally, absent predicates and the Class III status of breast implants imply a PMA pathway for any tissue-engineered breast device in the U.S., with point-of-care manufacturing frameworks still under FDA discussion [107,108,109,110,111]. Near-term trials should therefore prospectively capture quantitative volumetry, predefined complications (including seroma/revisions), device absorption status, and BREAST-Q outcomes to enable rational scale-up.

### 5.4. Future Prospects and Potential Impact of Tissue Engineering in Breast Reconstruction

In the evolving field of breast reconstruction, tissue engineering stands poised to revolutionize treatment paradigms. Harnessing the synergy of advanced biomaterials, 3D printing, and regenerative medicine, the prospect of creating personalized, biocompatible reconstructions that mimic the native breast tissue’s form and function is on the horizon. This transition promises enhanced aesthetic and functional outcomes and a potential reduction in post-surgical complications. By addressing current limitations and intricacies of traditional reconstructive procedures, tissue engineering could elevate the standard of care, offering patients natural-feeling results and enhancing the quality of life.

## 6. Conclusions

Tissue engineering and 3D printing technologies represent significant potential to address existing limitations in breast reconstruction following mastectomy. The integration of biodegradable biomaterials, such as PLA, PGA, PLGA, and P4HB, offers promising strategies to mimic the native structure and function of breast tissue, aiming for enhanced aesthetic and functional outcomes. However, critical gaps persist, notably in the biocompatibility, degradation kinetics, and biomechanical properties of these materials, as revealed by current preclinical evidence.

## Figures and Tables

**Table 2:** Evaluation of biodegradable materials in preclinical models: biocompatibility, degradation kinetics and biomechanical properties.

Material (References: [59,60,61,62,63,64,65,66,67,68,69,70,71,72,73,74,75,76,77,78,79])	Biocompatibility	Degradation Kinetics	Biomechanical Properties	Key Points and Considerations
PLA (Polylactic Acid)	Moderate; can trigger inflammatory responses due to acidic degradation products (lactic acid).	6 to 12 months	Good initial mechanical properties but tends to become brittle.	Widely utilized; concern about inflammation due to acidic degradation byproducts.
PGA (Polyglycolic Acid)	Good biocompatibility; broadly accepted in medical applications such as sutures.	Rapid degradation within weeks to months, breaking down into glycolic acid.	High initial strength, diminishes quickly due to rapid degradation.	Beneficial for short-term applications; degradation may be too rapid for prolonged structural support.
PLGA (Poly(lactic-co-glycolic acid)	Generally good; however, inflammatory concerns exist due to acidic degradation products.	Adjustable degradation time from weeks to months depending on the PLA to PGA ratio.	Mechanical properties adjustable through composition ratio (versatile).	Highly customizable; requires careful formulation to balance degradation rate and inflammatory response.
P4HB (Poly-4-hydroxybutyrate)	Excellent biocompatibility with minimal inflammatory response.	Degrades over approximately 12 to 18 months into 4-hydroxybutyric acid.	Flexible, robust mechanical strength suited for soft tissue implants.	Ideal for long-term, flexible support; more complex and costly due to exclusive fermentation-based synthesis.
Poly(D,L-lactide)	Moderate biocompatibility; inflammatory response potential similar to PLA.	Similarly to PLA; adjustable by altering blend ratio of stereoisomers.	Properties depend on stereoisomer ratios; can exhibit brittleness.	Mechanical and degradation profiles can be customized, yet inflammatory potential remains a concern.

**Table 3:** Clinical studies on reconstructive materials.

Author, Year	Study Objective	Methodology	Materials Used	Key Findings	Limitations
Rehnke, 2020 [92]	Evaluate effectiveness of composite strategy combining absorbable mesh with autologous fat grafting	Retrospective review, 22 patients, 28 reconstructed breasts, mean follow-up 19 months	Lotus scaffold (TIGR Matrix, SERI Scaffold, PHASIX mesh), Autologous fat graft	High elasticity, natural feel; histology: PHASIX mesh had superior fat structuring and milder foreign body response	Small sample size, retrospective design, limited follow-up period
Morrison, 2016 [93]	Assess clinical feasibility of TEC for adipose tissue growth	Case series, 5 patients, TEC with TAP flaps, follow-up up to 6–12 months	Acrylic chambers, thoracodorsal artery perforator (TAP) flaps	One patient achieved significant tissue expansion (210 mL); others no significant growth	Small sample size, limited success, patient discomfort led to early removal
Clinical trial NCT05460780 [94,95]	Safety and efficacy of bioabsorbable TEC with LICAp/LTAp flap	Ongoing trial, immediate reconstruction post-mastectomy	Bioabsorbable TEC, LICAp or LTAp pedicled flaps	Preliminary results: successful implantation in first human case (as reported)	Awaiting comprehensive data and long-term follow-up results
van Turnhout, 2018 [96]	Evaluate SERI surgical scaffold for direct-to-implant reconstruction	Retrospective review, 16 patients, 22 breasts; literature review included	SERI surgical scaffold	High complication rate (seroma 45%, scaffold integration issues 14%)	Retrospective, small sample, potential product-associated bias
Clinical trial NCT05437757 [97]	Safety and efficacy of fat grafting within 3D-printed scaffolds	Prospective trial, recruiting 20 participants	3D-printed polycaprolactone scaffold, autologous fat	Ongoing, preliminary safety and effectiveness assessment in progress	Awaiting results, small planned sample

## Data Availability

No new data were created or analyzed in this study. Data sharing is not applicable to this article.
